# Cost-Utility Analysis of Erenumab Compared to Topiramate for Preventive Therapy of Migraine in Iran

**DOI:** 10.5812/ijpr-146026

**Published:** 2024-09-16

**Authors:** Hosein Mollaee, Sadra Nadimi Parashkouhi, Behzad Fatemi, Meysam Seyedifar, Fatemeh Soleymani

**Affiliations:** 1 Department of Pharmacoeconomics and Pharmaceutical Administration, Faculty of Pharmacy, Tehran University of Medical Sciences, Tehran, Iran; 2 Pharmaceutical Management and Economics Research Center, Tehran University of Medical Sciences, Tehran, Iran

**Keywords:** Cost-Utility Analysis, Erenumab, Migraine, Model-Based Analysis, Topiramate

## Abstract

**Background:**

Migraine is a prevalent, chronic neurovascular disorder that incurs significant indirect costs due to productivity loss. Preventive therapy is an effective way to alleviate the societal and healthcare burden of migraine. Approximately 14% of both the global and Iranian populations are affected by migraine, which has substantial economic implications.

**Objectives:**

To determine the cost-effectiveness of Erenumab compared to Topiramate for migraine treatment in Iran.

**Methods:**

A three-state Markov model was used to evaluate the cost-effectiveness of Erenumab. The model considered both direct and indirect costs from a societal perspective. The incremental cost-effectiveness ratio (ICER) was calculated by determining the cost per quality-adjusted life year (QALY) gained. Costs and QALYs were discounted annually at 5.8% and 5%, respectively. Deterministic and probabilistic sensitivity analysis (PSA) were performed to assess the robustness of the model.

**Results:**

The average cost for patients using the Erenumab strategy was 16,836 USD over five years, whereas the average cost for the Topiramate strategy was estimated to be 2,660 USD. Additionally, the average QALYs for the Erenumab and Topiramate strategies were 3.64 and 3.46, respectively. The ICER for the Erenumab strategy was 78,923 USD/QALY. This ICER is significantly higher than the fixed Iranian willingness-to-pay (WTP) threshold of 2,456 USD.

**Conclusions:**

The study concludes that preventive treatment of migraine with Erenumab, compared to Topiramate, is not cost-effective in Iran based on current prices. Therefore, for Erenumab to be considered cost-effective, a significant price reduction is necessary for its entry into the Iranian pharmaceutical market.

## 1. Background

Migraine is a common, chronic neurovascular disorder typically accompanied by headaches, pain, nausea, vomiting, and sensitivity to light and sound (1). Approximately 14% of the global and Iranian populations are affected by migraine (1, 2), which has a significant economic impact on society. Migraine is frequently reported in certain demographics, particularly among women and individuals aged 35 to 45 (2).

Migraines affect young people and result in significant indirect costs to society due to productivity loss. Patients with migraines also incur increased annual direct medical expenses compared to matched individuals without migraines. For example, the average annual cost for medical services among medication overuse headache (MOH) patients in Iran is $1,046, with an additional $132 for nonmedical services and $1,432 attributed to lost productivity (3).

Treatment options for migraine are classified into acute or abortive treatment and prophylactic or preventive treatment. Managing acute migraine attacks often involves the use of analgesics, nonsteroidal anti-inflammatory drugs (NSAIDs), and triptans, which aim to stop the progression of a headache in the early hours. Preventive treatment aims to decrease the frequency of attacks, enhance responsiveness to the severity and duration of acute attacks, and minimize disability (4). Primary pharmacological therapies for migraine prevention include beta-blockers, anticonvulsants, antidepressants, and calcitonin gene-related peptide (CGRP) therapy. The European Headache Federation recommends considering monoclonal antibodies targeting the CGRP pathway as a primary choice for preventive treatment in individuals with migraines (5).

Erenumab is the only FDA-approved fully human monoclonal antibody targeting the CGRP receptor for the prevention of migraine in adults with a minimum of four monthly migraine days (MMDs) (6). Post hoc analysis of data from pivotal studies in patients with both episodic migraine (EM) and chronic migraine (CM) supports the efficacy of Erenumab at 70 mg or 140 mg doses, demonstrating a reduction in the number of MMDs (7, 8).

Topiramate is an antiepileptic medication that is FDA-approved for the prevention of migraine (9). It is the most common first-line therapy option in international guidelines (10) and has been studied more extensively than other migraine preventive drugs in clinical trials (11). According to two randomized, double-blind, placebo-controlled clinical trials, the optimal dosage of Topiramate for migraine prevention is 100 mg per day (12, 13). Topiramate is widely recommended as a first-line therapy in international guidelines, such as the American Academy of Neurology/American Headache Society (AAN/AHS) evidence-based guidelines and the European Federation of Neurological Societies (EFNS) clinical guidelines, which classify Topiramate as a proven effective migraine prophylactic agent with a grade A recommendation (14, 15). Additionally, Topiramate is one of the most available and affordable migraine-preventive medications in Iran.

## 2. Objectives

Given the disease and economic burden of migraine in Iran, along with the high efficacy and tolerability of Erenumab, a novel CGRP antibody, this study aims to compare its cost-effectiveness profile with that of Topiramate for migraine prevention in Iran. We hope that the findings of this study will provide sufficient evidence to inform healthcare decision-making in Iran, a middle-income country with a high prevalence of migraine.

## 3. Methods

### 3.1. Study Characteristics

A model-based cost-utility analysis was conducted to evaluate the cost-effectiveness profile of Erenumab compared to Topiramate in managing episodic and chronic migraines in Iran. The model included a hypothetical population of 1,000 patients, with 88.9% suffering from episodic migraine and 11.1% from chronic migraine, based on the Reuter et al. study (16). Individuals with 0 to 14 MMDs are classified as having episodic migraine, while those with 15 or more MMDs are classified as having chronic migraine (17). The outcome of therapeutic interventions, measured by reducing MMDs, was expressed in terms of quality-adjusted life years (QALYs). The study also considered all direct and indirect costs from a societal perspective over a five-year time horizon, with 60 monthly treatment cycles. The resulting incremental cost-effectiveness ratio (ICER) was evaluated using a fixed Iranian willingness-to-pay (WTP) threshold of 2,456 USD/QALY (18). All aspects of this study were conducted and reported according to the CHEERS checklist (Appendices).

### 3.2. Model Structure

**Figure 1.**
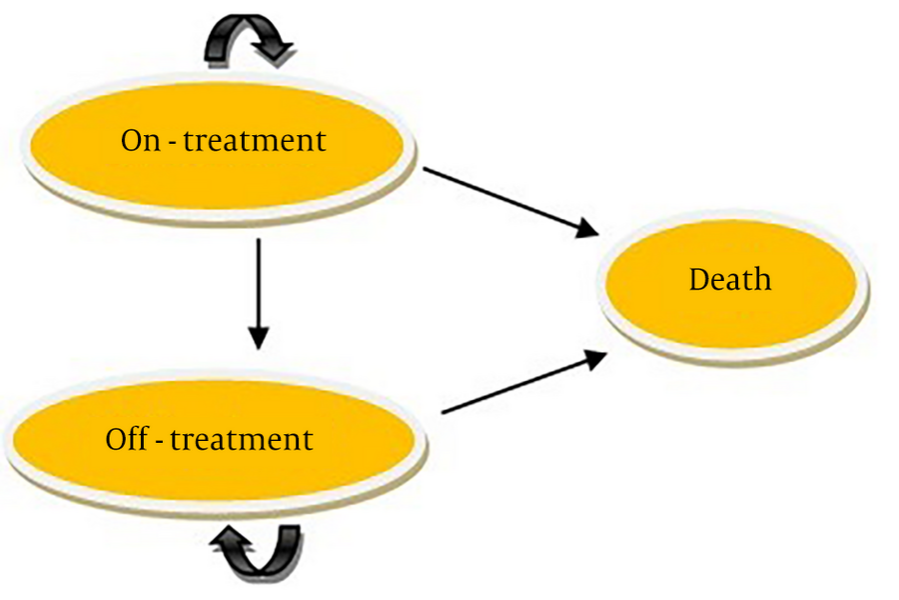
Model structure

The study employed a three-state Markov model, consisting of on-treatment, off-treatment, and death states, to simulate patient cohorts and assess outcomes (Figure 1). Patients had the option to continue or discontinue treatment if they experienced intolerable adverse effects (AEs). In each state, patients faced the same probability of age-standardized natural death rates. Patients who continued treatment enjoyed a better quality of life compared to those who stopped due to AEs. In the off-treatment state, patients received standard of care (SOC) treatment rather than Erenumab or Topiramate. To simplify the model, it was assumed that patients in the off-treatment state did not receive any preventive treatment and used only palliative therapy for migraine attacks at the same rate as on-treatment patients experiencing migraine attacks. The cycle length was 28 days. Over a five-year time horizon, the model estimated total and incremental health outcomes, total and incremental costs for each intervention, and the ICER. Since the study time horizon exceeded one year, costs and QALYs were discounted annually at rates of 5.8% and 5%, respectively. The model was constructed using TreeAge Pro software (version 2022). Conventional half-cycle corrections were applied throughout the modeling phase. 

### 3.3. Data Collection

Information on quality of life, costs, and the probability of clinical events was gathered from the literature. Specifically, data on MMDs, medication utilization rates, patients' quality of life, the effectiveness and AEs of each comparison arm, and treatment discontinuation rates were prerequisites for the study.

Electronic databases, including PubMed, Scopus, Cochrane, and Web of Science, were systematically reviewed up to May 2023 to capture clinical data. The search strategies and screening results of the systematic review can be found in the Appendices. Additionally, the findings of the systematic review were validated through consultation with experts. The HER-MES study, a randomized double-blind controlled trial involving EM and CM patients, was selected as the reference study. Thus, the baseline characteristics and outcomes of the modeled population were determined based on the HER-MES study (16).

### 3.4. Probabilities

The probability of patients transitioning between different health states was calculated using the rate-to-probability conversion formula: p(t) = 1 - e^-rt^ where p represents the probability, t is time, e is the mathematical constant (2.718), and r is the rate. The collected rates included baseline MMD, the number of MMDs in both preventive strategies and off-treatment, Erenumab and Topiramate withdrawal rates over 6 months, and the natural age-standardized mortality rate for the Iranian population (19). All probabilities were calculated for 30-day intervals (Supplementary File).

### 3.5. Outcomes

The primary outcome of the study was QALYs, which were calculated based on changes in patients’ health-related quality of life (HRQOL). Health-related quality of life was assessed using the reduction in MMDs and adverse pharmaceutical effects observed during the HER-MES study (16).

Since migraine does not directly affect life expectancy, we assumed that the mortality rate in both study arms was equal to the natural age-standardized mortality rate for the Iranian population (19). Based on Xu et al. (20), HRQOL values for days with and without migraine attacks were set at 0.44 and 0.933, respectively. It was also assumed that patients who discontinue migraine preventive medication do not switch to other studied alternatives but return to the SOC.

According to research by Matza et al. (21) and Reuter et al. (16), the incidence of serious AEs leading to treatment discontinuation and their impact on patients' quality of life were significant, as described in Table 1. After consulting with an expert panel, it was assumed that in the case of serious AEs, patients would experience these complications for one week. Consequently, a one-week disutility was applied for patients experiencing serious AEs.

**Table 1.** Disutility Values

Adverse Effect	Adverse Effect Rate (%)		Disutility	Disutility for 1 Week
	Erenumab	Topiramate		
**Fatigue**	2.3	7.5	-0.06	-0.0011
**Dizziness**	1	5.4	-0.01	-0.0002
**Paresthesia**	0	9.8	-0.012	-0.0002
**Attention deficit**	9.3	1.8	-0.098	-0.0019

### 3.6. Costs

Cost estimation was performed from a societal perspective using a micro-costing method. We considered all direct medical and non-medical costs, as well as indirect costs. To determine these costs, we reviewed guidelines, related studies, expert interviews, and medical records. The cost for each health status was calculated by multiplying the quantity of each item used in a cycle by its price. Prices for services and medicines were obtained from the latest edition of the relative values of health services book (22, 23). Additionally, to calculate indirect costs, productivity loss due to migraine days was estimated based on the minimum wage in Iran in 2023.

Since no medication was shown to have high-risk common AEs in the reference trial, those costs were not considered, and it was assumed that medication would be discontinued if AEs were intolerable. The share of private and public specialist visits was estimated at 35% and 65%, respectively, with visit prices calculated based on registered tariffs (22). All costs were reported in US dollars, using the government exchange rate of 1 USD = 285,000 IR Rial (18).

### 3.7. Parameter Distributions

The distribution of the parameters is shown in Table 2, which details the mean values and types of distributions.

**Table 2.** Model Inputs

Input	Values	Distribution
**Cost ($)**		
Oral acute medication	0.12	-
Parental acute medica-tion	2.48	-
Erenumab 70 mg	290	-
Erenumab 140 mg	348	-
Topiramate 100 mg	0.06	-
General practitioner vis-it	1.79	-
Specialist physician vis-it	3.28	-
Cost of injection	1.50	-
Direct nonmedical (trip)	0.70	-
Productivity loss (per each working day)	6.21	-
**Rates and probabilities ** ^**a**^		
Mean baseline MMD	10.4 ± 3.9	Normal
Mean number of MMD		
Erenumab	4.54 ± 2.18	Log normal
Topiramate	6.38 ±1.82	Log normal
Off treatment	10.4 ± 3.9	Log normal
Withdrawal rate (in 6 months)		
Erenumab	10.6% ± 4.3	Log normal
Topiramate	38.9% ± 14.4	Log normal
**Annual health related quality of life ** ^**a**^		
Baseline	0.76 ± 0.08	Beta
Off treatment	0.76 ± 0.08	Beta
On treatment		
Erenumab	0.86 ± 0.085	Beta
Topiramate	0.83 ± 0.079	Beta

Abbreviation: MMD, monthly migraine day.

^a^ Values are expressed as mean ± SD.

### 3.8. Sensitivity Analysis

Deterministic and probabilistic sensitivity analysis (PSA) were conducted to assess uncertainties related to the model and the evidence obtained from it.

## 4. Results

### 4.1. Base Case Cost-effectiveness Analysis

According to the base case analysis, the average cost for the Erenumab and Topiramate strategies was $16,836 and $2,660 per patient over five years, respectively. Additionally, within this time horizon, the average QALYs for these two alternatives were 3.64 and 3.46, respectively. The ICER was calculated at $78,923, which, when compared to the Iranian WTP threshold of $2,456, does not support the cost-effectiveness of the Erenumab strategy. This indicates that Erenumab is not cost-effective compared to Topiramate for managing migraine in Iran (Table 3). 

**Table 3.** Cost-effectiveness of Erenumab vs. Topiramate

Strategy	Cost (USD)		Effect (QALY)		ICER	Net Monetary Benefit
	Cost	Incremental Cost	Effect	Incremental Effect		
**Topiramate**	2,660		3.46			69,476
**Erenumab**	16,836	14,176	3.64	0.18	78,923	59,044

Abbreviations: QALY, quality-adjusted life year; ICER, incremental cost-effectiveness ratio.

### 4.2. Sensitivity Analysis

**Figure 2.**
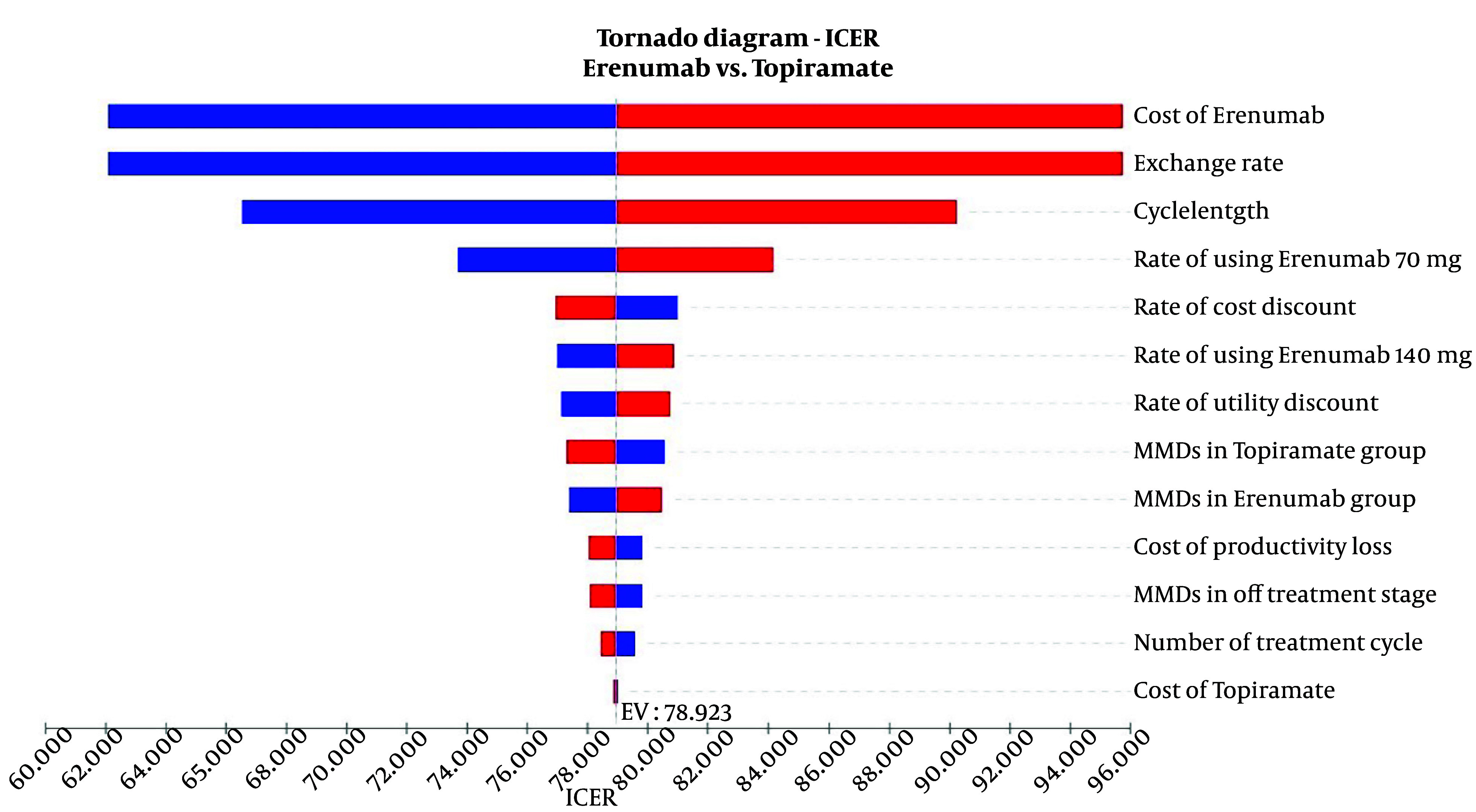
Tornado diagram

**Figure 3.**
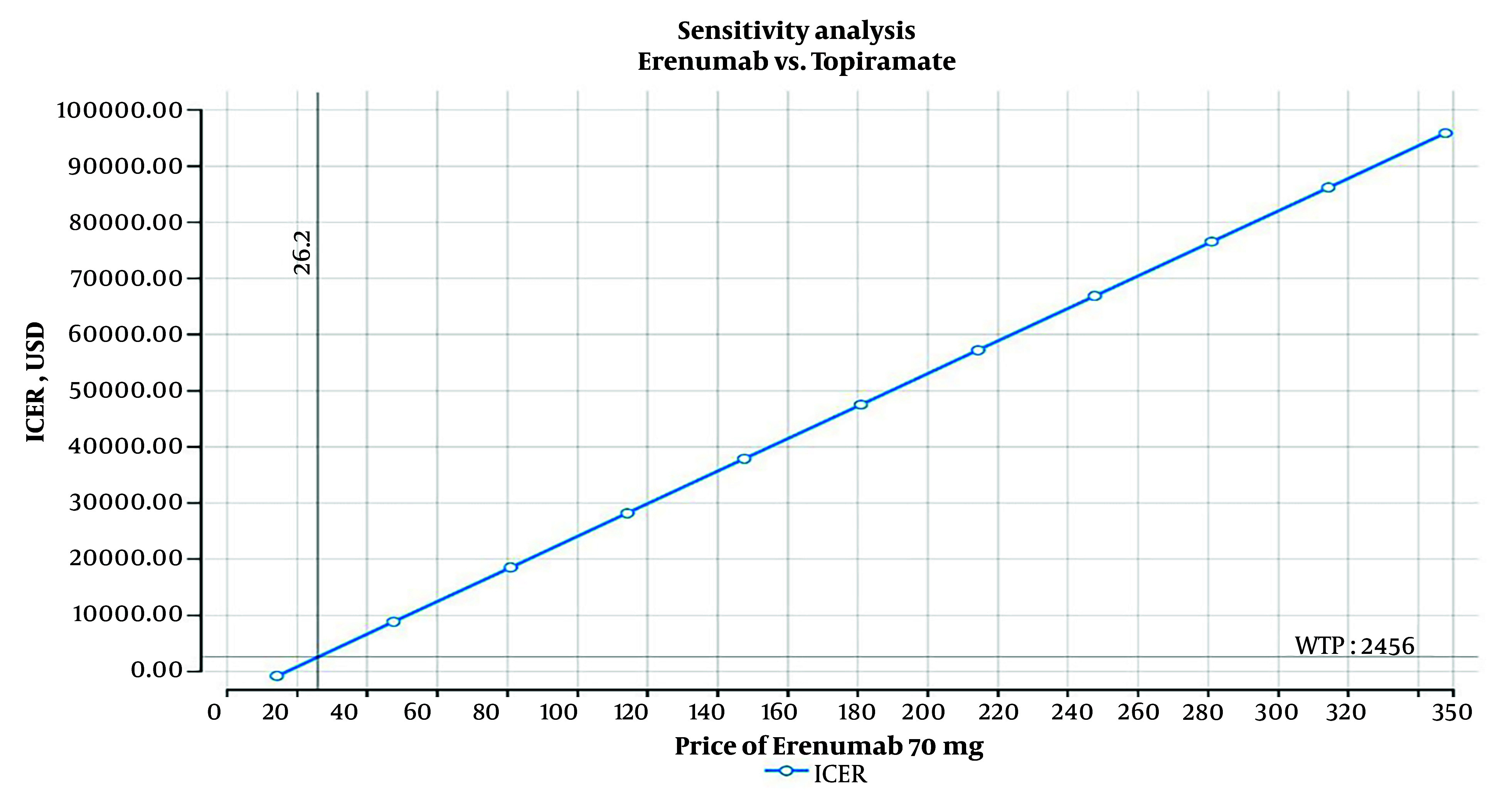
One-way sensitivity analysis

The findings of the one-way sensitivity analysis are presented in Figures 2 and 3. According to the Tornado plot, none of the variables considered have a significant impact on the main model results, as they vary within ± 20% (Figure 2). This indicates that the model remains stable within this range of changes. Consequently, the ICERs obtained do not exceed Iran's cost-effectiveness threshold of $2,456, as determined by Iran's Food and Drug Organization (18).

The one-way sensitivity analysis for Erenumab indicated that this medication can be cost-effective in Iran only if priced below $26.20. However, this price is significantly lower than the minimum cost of $90 for Erenumab in reference countries' markets (Figure 3). 

**Figure 4.**
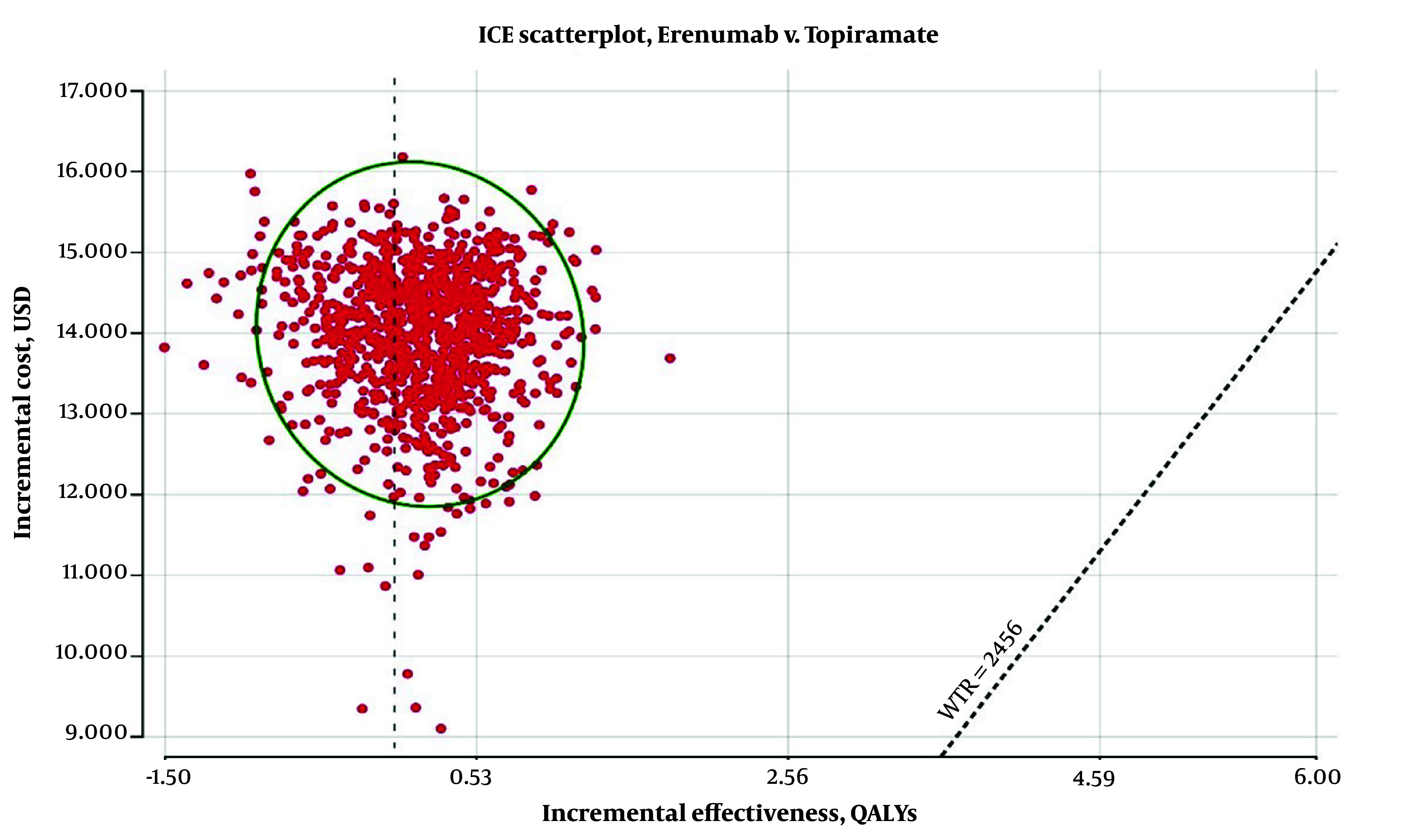
Incremental cost-effectiveness ratio (ICER) scatterplot of Erenumab vs. Topiramate

Finally, as shown in Figure 4, the results of the PSA demonstrate that Erenumab, priced at $290 per dose, cannot be cost-effective in more than 1% of cases compared to Topiramate.

## 5. Discussion

This study represents the first economic evaluation comparing the cost-effectiveness of Erenumab and Topiramate. It is also the first to incorporate data from a head-to-head study between a CGRP pathway-targeting antibody and Topiramate. The results of the HER-MES study indicate that Erenumab treatment is associated with superior tolerability and significantly greater efficacy compared to Topiramate. Consequently, preventive therapy with Erenumab leads to an improvement in quality of life compared to Topiramate.

Our study found that the costs of treatment with Erenumab and Topiramate are $16,836 and $2,660, respectively, based on a drug price of $290 for 70 mg of Erenumab, which is the price in reference countries. From a societal perspective, the ICER for preventive therapy with Erenumab versus Topiramate is $78,923. Erenumab is not cost-effective given the WTP threshold of $2,456 in Iran. Additionally, deterministic sensitivity analysis showed that the model results are not significantly affected by any specific parameter within the variation range of ±20%. Moreover, Erenumab is only a cost-effective alternative in less than 1% of PSA results.

Although Erenumab showed better efficacy in the HER-MES study and in our model-based analysis, it appears that, at the price available in reference countries, Erenumab is not a cost-effective option in Iran compared to Topiramate. For Erenumab to be considered cost-effective, a price reduction of over 90% is required.

Erenumab received its first approval in 2018. Since then, several studies have evaluated the cost-effectiveness of this drug and other CGRP pathway-targeting antibodies for migraine prevention.

Sussman et al. (2) assessed the cost-effectiveness of Erenumab compared to no treatment or OnabotulinumtoxinA in patients who had previously failed preventive therapy, considering both US societal and payer perspectives. Their study, using a hybrid Monte Carlo patient simulation and Markov cohort model, found that Erenumab is cost-effective for reducing monthly migraine days from a societal perspective but not from a payer perspective. Giannouchos et al. (1) performed a cost-effectiveness analysis in Greece using a decision-tree model, and found that Erenumab is not cost-effective compared to OnabotulinumtoxinA. Their analysis suggested that Erenumab could be considered cost-effective at a threshold price below €192 (societal perspective) or €173 (payer perspective). Additionally, Mahon et al. (6) determined the cost-effectiveness of Erenumab compared with the best supportive care for patients who had failed at least two prior preventive treatments. They found that preventive therapy with Erenumab results in ICERs of €3,310 per QALY gained, making it a cost-effective treatment for migraine prevention from a societal perspective with a WTP threshold of €28,528 per QALY.

There are certain differences in how our study was designed compared to earlier studies. Firstly, we combined the results for episodic and chronic migraine groups since the clinical trial did not provide separate data for EM and CM patients. Secondly, we assessed HRQOL by measuring the average change in MMDs, as we could not access reliable HRQOL data from questionnaires.

Considering these factors, Erenumab may be a cost-effective option only in high-income countries with a societal perspective at current prices. Therefore, it appears that Erenumab, at its current price, is not a cost-effective choice for Iran, a lower-middle-income country.

When using or generalizing the findings of this study, it is important to consider some limitations. Specifically, we had to rely on literature data due to a lack of local clinical data and the local domestic market price of Erenumab. To obtain HRQOL estimates, the HER-MES study collected data using the HIT-6, a disease-specific survey instrument. However, the HIT-6 mapping algorithm had a low R^2^ score and was erratic; as a result, utility data were derived from other studies in which HRQOLs were evaluated using EuroQol-5 dimension (EQ-5D) based on the number of MMDs and AEs associated with Topiramate and Erenumab. Additionally, treatment discontinuation rates were considered only for the first six months of the study.

Our study concludes that Erenumab, at its current global market price, is not a cost-effective option compared to Topiramate for treating migraines in Iran. However, we determined that a significant price reduction (approximately 90%) would be necessary for Erenumab to be considered cost-effective and eligible for entry into the Iranian pharmaceutical market.

ijpr-23-1-146026-s001.pdf

## Data Availability

The dataset presented in the study is available on request from the corresponding author during submission or after publication.
