# Supplementary material for: Cost-Utility Analysis of Erenumab Compared to Topiramate for Preventive Therapy of Migraine in Iran
Source: Iran J Pharm Res. 2024 Sep 16;23(1):e146026. doi: 10.5812/ijpr-146026 (PMC11742578; doi:10.5812/ijpr-146026)
Supplement: ijpr-23-1-146026-s001.pdf [file ijpr-23-1-146026-s001.pdf]

Supplementary material 1:

1. Model inputs

The following Table lists the price of topiramate, together with the cost of all medications and equipment needed during a migraine attack, based on the most recent prices accessible by the Iran Food and Drug Organization.

Table 1. Medicine and medical equipment costs

Medicine	Unit price (USD)
Topiramate Tablet Oral 100mg	0.06
Sumatriptan Tablet Oral 50mg	0.09
Sumatriptan Tablet Oral 100mg	0.17
Rizatriptan Tablet Oral 5mg	0.10
Rizatriptan Tablet Oral 10mg	0.20
Rizatriptan (As Benzoate) Tablet, Orally Disintegrating Oral 10mg	0.26
Ibuprofen Capsule, Liquid Filled Oral 400mg	0.07
Acetaminophen Tablet 500mg	0.02
Acetaminophen / Caffeine / Ibuprofen Capsule Oral 325mg/40mg/200mg	0.06
Ergotamine Tartrate / Caffeine Tablet Oral 1mg/100mg	0.04
Acetaminophen / Codeine Phosphate Tablet Oral 300mg/20mg	0.02
Acetaminophen 1g/100 ml Inj	0.63
Dexamethasone 8mg/2 ml Amp	0.25
Ketorolac 60mg/2 ml Inj	0.20
Ondansetron HCl 4mg/2 ml Amp	0.22
Dextrose Nacl 3.33% 0.3% 0.5 l Inf	0.76
Infusion Set	0.45
Scalp	0.10
Syring 3 ml	0.09

Syring 5 ml	0.10

Since Erenumab is not available in IDL, its price was set using the prices of reference countries: Spain and South Africa were the only two countries where this medication was available, and these two countries' prices were used to determine the price of this medication, as shown in the Table .

Table 2. Erenumab cost

Medicine	Price of medicine in Spain	Price of medicine in South Africa
Erenumab 70 mg	515.75 €	290 $
Erenumab 140 mg	Not available	Not available

Considering the lower price of medicine in South Africa, it was used as a reference price in the calculations. Since the price of Erenumab 140 mg was not available in the reference countries, it was calculated according to the ratio of the price of the dose of 140 mg to the price of the dose of 70 mg in other countries (which was about 1.2) and the figure of 348 USD was determined.

The cost of outpatient hospitalization in clinics, in case of no improvement, has been calculated according to the rate of referral to medical centers obtained from the studies and the information obtained based on the tariffs announced by the Ministry of Health and the prices of medicines consumed.

We assumed that the person had a function on the day of his migraine and calculated one day of absence from work for each day of migraine as the study's perspective was from society. The national average salary for a single working day was used to compute the cost of one working day. According to the Iran Ministry of Labor and Social Welfare, the lowest monthly salary and cost of each working day are 263.79 (USD) AND 8.79 (USD) respectively. Based on the average taxi fare for a 2- to 5-kilometer trip, the anticipated cost of transportation to the outpatient treatment center is 0.70 USD.

Table 3. Medicines used in the emergency state

	Medicine
1	Acetaminophen 1 G/100 mL INJ
2	Dexamethasone 8 mg/2 mL AMP
3	Ketorolac 60 mg/2 mL INJ
4	Ondansetron HCl 4 mg/2 mL AMP
5	Dextrose NaCl 3.33% 0.3% 0.5l INF

Table 4.The most often used painkillers in Iran [1].

Medicine category	Use (%)	Most used in Iran
Triptans	32.8	− Sumatriptan Tablet Oral 50mg<br>− Sumatriptan Tablet Oral 100mg<br>− Rizatriptan Tablet Oral 5mg<br>− Rizatriptan Tablet Oral 10mg<br>− Rizatriptan (As Benzoate) Tablet, Orally Disintegrating 10mg
Analgesics	33.3	− Ibuprofen Capsule, Liquid Filled Oral 400mg<br>− Acetaminophen Tablet 500mg
Combination analgesics	16.3	− Acetaminophen / Caffeine / Ibuprofen Capsule Oral 325mg/40mg/200mg
Ergotamine	0.6	− Ergotamine Tartrate / Caffeine Tablet Oral 1mg/100mg
Opioids	0.5	− Acetaminophen / Codeine Phosphate Tablet Oral 300mg/20 mg
Combination therapy	16.5	− Acetaminophen / Caffeine / Ibuprofen Capsule Oral 325mg/40mg/200mg + Triptan

As triptans are a specialized medication used to treat migraine, the consumption of each of their multiple doses is derived using Iran's most recent medicine statistics, which date back to the year 2021 [2].This is detailed in the table 5.

Table 5. Triptans use in Iran based on 2021 statistics

Triptans	Use (%)
Sumatriptan Tablet Oral 50mg	59.9
Sumatriptan Tablet Oral 100mg	21.1
Rizatriptan Tablet Oral 5mg	5.2
Rizatriptan Tablet Oral 10mg	9
Rizatriptan (As Benzoate) Tablet, Orally Disintegrating 10mg	4.8

Since Ibuprofen and Acetaminophen were not specifically designed to treat migraine, it is impossible to determine the appropriate dosage for them based on statistics. Therefore, it was considered that 50% of each medication would be taken after seeing a specialist.

Outcomes

The following **Error! Reference source not found.** details the extent to which each of the two medications responded over a four- to six-month period:

Table 6. Efficacy of topiramate and erenumab

	**Erenumab (388)**	**Topiramate (388)**
> 50% Reduction in migraine days per month (%)	215 (55.4)	121 (31.2)
Change in number of migraine days (Mean)	-5.86	-4.02

As can be seen in the **Error! Reference source not found.** below, the topiramate group experienced a significantly greater rate of drug adverse effects compared to the Erenumab group, which caused the patient to discontinue the medication completely.

Table 7. Rate of AEs of erenumab and topiramate

**Side effect**	**Erenumab (388)**	**Topiramate (388)**
Side effects that cause discontinuation (%)	41 (10.6)	151 (38.9)
Paresthesia (%)	0 (0)	38 (9.8)
Attention deficit (%)	7 (1.8)	36 (9.3)
Fatigue (%)	9 (2.3)	29 (7.5)
Dizziness (%)	4 (1)	21 (5.4)

The dosage for each patient was established based on the results of the clinical trial; About 65% of participants started the medication at a dose of 140 mg per month, or they started at a dose of 70 mg and increased it to 140 mg per month. 35% of participants consumed a dose of 70 mg per month from the start of the study to the end. For topiramate, the patients were given an average of 91 mg of the medication each day, which was included in the cost estimation as the average cost for one 100 mg pill. Finally, the conversion of the mentioned outcomes to QALY was done as mentioned in the method.

Probabilities

All potential outcomes in this study should be expressed in terms of a single migraine day, as the difference in the reduction of the number of migraine days was examined in the clinical trial. According to a prior national study, the average number of migraine days for patients with chronic migraine was 21 days, and for patients with periodic migraine, it was 6 days [3].

Regarding the hospitalization rate of patients in Iran, since injections are performed in the clinics, there are significantly fewer emergency visits and hospitalizations for migraine attacks than in other nations; additionally, since the hospitalization rate for migraine patients in Iran was unknown, it was not taken into account in this study, and the rate of physician visits was derived from the overall rate of physician visits, emergency room visits, and hospitalizations in other nations, as reported by Hallier et al. [4] which is shown in the **Error! Reference source not found.**.

Table 8. The number of visits to medical centers over 12 weeks

	**Physician visit**	**Emergency visit**	**Hospitalization**
**Periodic migraine**	0.69	0.1	0.03
**Chronic migraine**	2.07	0.41	0.09

The probability of visiting medical centers for each migraine attack was calculated as below:

Periodic migraine: (0.69+0.1+0.03)/3/6=0.45

Chronic migraine: (2.07+0.41+0.09)/3/21=0.40

Since patients with chronic and periodic migraines have very similar probabilities of visiting medical centers for each attack, the probability of visiting medical centers for periodic patients was used for both groups.

Assuming that the patient will take painkillers for every episode of migraine, the percentage of painkillers and anti-migraine medications was determined in the model based on pre-studies and consultation with an expert. The most often used painkillers in Iran were chosen after consulting with an expert as there was no data available regarding the use of any of the pharmaceuticals in the aforementioned medicine categories. These medicines include Triptans, Analgesics, Ergotamine, Opioids, and Combination therapies. Further information regarding the use of mentioned medicines is displayed in the supplementary file.

Figure and Figure , describe the transition probability of staying on treatment, discontinuing, and death state in each strategy based on selected patient cohort.

Rate to probability conversion formula:

$$\text{Probability} = 1 - \exp(-\text{rate})$$

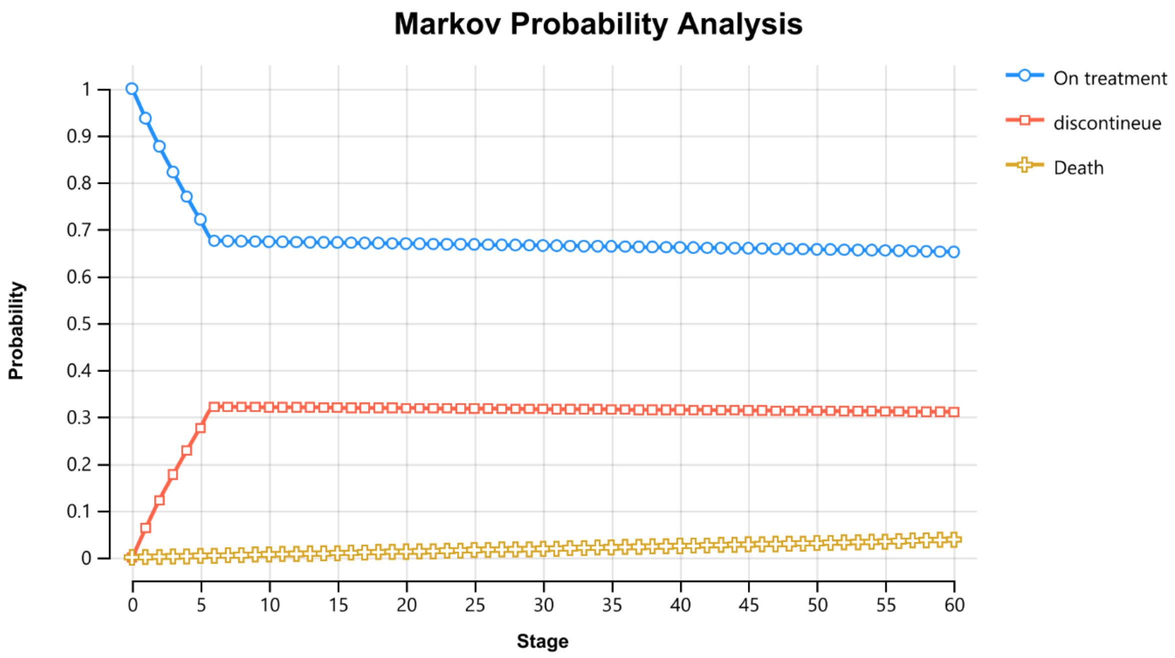

Figure 1. Topiramate probability analysis

Table 9. Probability of patients transition between different health states in topiramate group in first 6 month

	On treatment	Off treatment	Death
On treatment	0.93237	0.067	0.00063
Off treatment	0	0.99937	0.00063
Death	0	0	1

Table 10. Probability of patients transition between different health states in topiramate group after first 6 month

	On treatment	Off treatment	Death
On treatment	0.99937	0	0.00063
Off treatment	0	0.99937	0.00063
Death	0	0	1

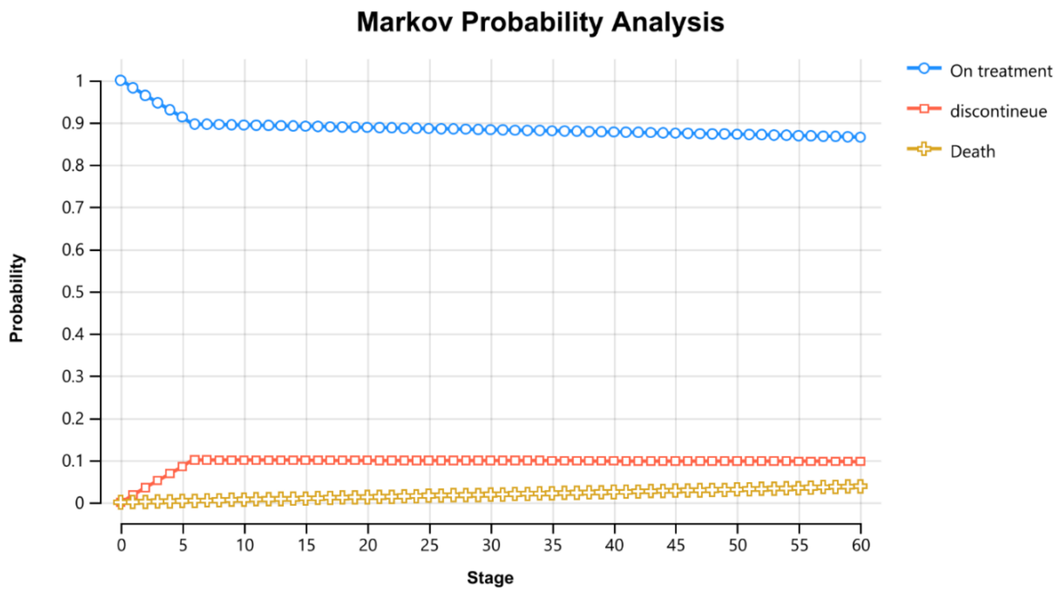

Figure 2. Erenumab probability analysis

Table 11. Probability of patients transition between different health states in erenumab group in first 6 month

	On treatment	Off treatment	Death
On treatment	0.98037	0.019	0.00063
Off treatment	0	0.99937	0.00063
Death	0	0	1

Table 12. Probability of patients transition between different health states in erenumab group after first 6 month

	On treatment	Off treatment	Death
On treatment	0.99937	0	0.00063
Off treatment	0	0.99937	0.00063
Death	0	0	1

In the phase 4 trial by Reuter et al., the treatment withdrawal followed an exponential pattern until week 16. Afterward, the withdrawal rate remained constant for both groups. To account for this trend, the current study assumes that intolerable complications will manifest by the 16th week [5].

2. Sensitivity analysis

The result of the Two-way sensitivity analysis can be found in **Error! Reference source not found.**. The topiramate strategy is considered cost-effective in most cases except when the price of Erenumab falls below 26.2 USD.

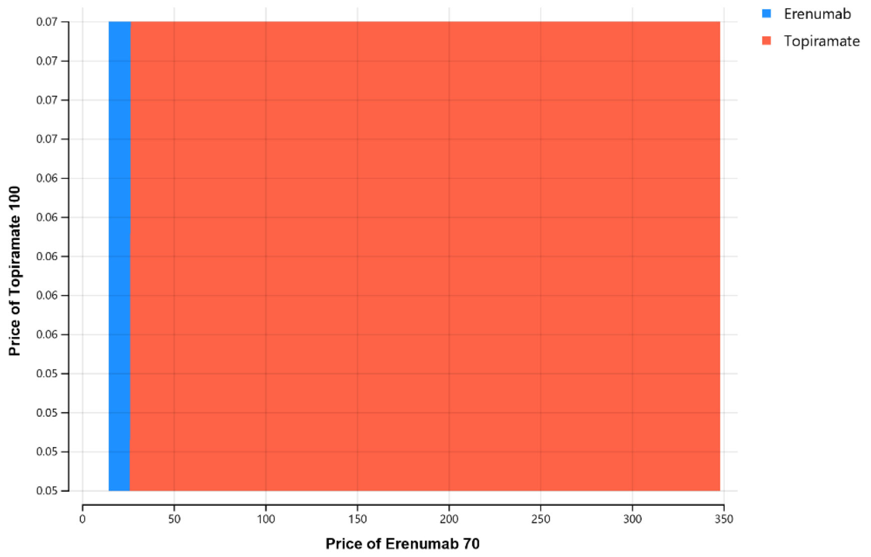

Figure 3. Two-way sensitivity analysis

Table 13. ICER frequency

COMPONENT	QUADRANT	INCREFF	INCRCOST	INCRCE	FREQUENCY	PROPORTION
**C1**	IV	IE>0	IC<0	Superior	0	0
**C2**	I	IE>0	IC>0	ICER<2456.0	0	0
**C3**	III	IE<0	IC<0	ICER>2456.0	0	0
**C4**	I	IE>0	IC>0	ICER>2456.0	675	0.675
**C5**	III	IE<0	IC<0	ICER<2456.0	0	0
**C6**	II	IE<0	IC>0	Inferior	325	0.325
**Indiff**	origin	IE=0	IC=0	0/0	0	0

1. Cowan, R.P., et al., Quantity changes in acute headache medication use among patients with chronic migraine treated with eptinezumab: subanalysis of the PROMISE-2 study. The Journal of Headache and Pain, 2022. 23(1): p. 115.

2. ; Available from: www.fda.gov.ir.

3. Togha, M., et al., Economic burden of medication-overuse headache in Iran: direct and indirect costs. Neurol Sci, 2021. 42(5): p. 1869-1877.

4. Hollier-Hann, G., et al., Updated cost-effectiveness analysis of onabotulinumtoxinA for the prevention of headache in adults with chronic migraine who have previously received three or more preventive treatments in the UK. J Med Econ, 2020. 23(1): p. 113-123.

5. Reuter, U., et al., Erenumab versus topiramate for the prevention of migraine - a randomised, double-blind, active-controlled phase 4 trial. Cephalalgia, 2022. 42(2): p. 108-118.
